# Supplementary material for: A Continuous Flow-through Microfluidic Device for Electrical Lysis of Cells
Source: Micromachines (Basel). 2019 Apr 13;10(4):247. doi: 10.3390/mi10040247 (PMC6523307; doi:10.3390/mi10040247)
Supplement: Supplementary file 1 [file micromachines-10-00247-s001.zip › micromachines-485999-Supplementary Materials/micromachines-485999-Supplementary.pdf]

micromachines

Supplementary Materials: Detailed Description of the Theory

A Continuous Flow-through Microfluidic Device for Electrical Lysis of Cells

Ying-Jie Lo and U Lei *

Institute of Applied Mechanics, National Taiwan University, Taipei 10617, Taiwan; d93543008@ntu.edu.tw
* Correspondence: leiu@iam.ntu.edu.tw; Tel.: +886-2-3366-5673

(I) Primary Flow

As the length of the microchannel in the present experiment is of two orders greater than its width and height and the Reynolds number is of order unity, the primary flow in the microchannel is the Poiseuille flow, the fully-developed laminar flow in a rectangular channel, with velocity components [26] (refer to reference [26] of the paper, and similar for later citations in this file)

$$u=\frac{4a^2}{\mu\pi^3}\left(-\frac{dp}{dx}\right)\sum_{i=1,3,5,\ldots}^{\infty}(-1)^{(i-1)/2}\left\{1-\frac{\cosh\left[i\pi\left(z-b/2\right)/a\right]}{\cosh\left[i\pi(b/2)/a\right]}\right\}\frac{\cos\left[i\pi\left(y-a/2\right)/a\right]}{i^3},\quad v=0,\quad w=0,\quad \text{(S1)}$$

and the volume flow rate

$$Q=\frac{ba^3}{12\mu}\left(-\frac{dp}{dx}\right)\left[1-\frac{192a}{\pi^5 b}\sum_{i=1,3,5,\ldots}^{\infty}\frac{\tanh\left[i\pi(b/2)/a\right]}{i^5}\right],\qquad \text{(S2)}$$

in terms of the notations in Figure 1a of the paper. Here (u, v, w) are the velocity components in the (x, y, z) coordinates, dp/dx is the pressure gradient along the x-direction, μ is the dynamic viscosity of the fluid (which is taken as 1.2 cP here for the mixture of whole blood and PBS solution). The stress components are:

$$\tau_{zx}=\tau_{xz}=\mu\frac{\partial u}{\partial z}=\frac{48\mu Q}{\pi^2 a^2 bD}\sum_{i=1,3,5,\ldots}^{\infty}\left(-1\right)^{\frac{i+1}{2}}\frac{\sinh\left[i\pi\left(z/a-0.5b/a\right)\right]\cos\left[i\pi\left(y/a-0.5\right)\right]}{i^2\cosh\left(0.5i\pi b/a\right)}\qquad \text{(S3a)}$$

and

$$\tau_{yx}=\tau_{xy}=\mu\frac{\partial u}{\partial y}=\frac{48\mu Q}{\pi^2 a^2 bD}\sum_{i=1,3,5,\ldots}^{\infty}\left(-1\right)^{\frac{i+1}{2}}\left\{1-\frac{\cosh\left[i\pi\left(z/a-0.5b/a\right)\right]}{\cosh\left(0.5i\pi b/a\right)}\right\}\frac{\sin\left[i\pi\left(y/a-0.5\right)\right]}{i^2},\quad \text{(S3b)}$$

with all other components zero. Here

$$D=1-\frac{192a}{\pi^5 b}\sum_{i=1,3,5,\ldots}^{\infty}\frac{\tanh\left(0.5i\pi b/a\right)}{i^5}.\qquad \text{(S4)}$$

The maximum magnitude of the mechanical shear stress, $\tau_{\max}$, occurs at the locations (x, 0.5a, 0) and (x, 0.5a, b) in Figure 1a, for the present device, and

$$\tau_{\max} = \tau_{xz}\big|_{(x,0.5a,0)} = -\tau_{xz}\big|_{(x,0.5a,b)} = \frac{48\mu Q}{\pi^2 a^2 bD} \sum_{i=1,3,5,...}^{\infty} (-1)^{(i-1)/2} \frac{\tanh(0.5i\pi b/a)}{i^2}. \quad \text{(S5)}$$

For a given volume flow rate Q, dp/dx can be estimated through Equation (S2), and thus u, τ_{zx}, τ_{yx} and $\tau_{\max}$, can be calculated using Equations (S1), (S3a), (S3b), and (S5), respectively.

In real situation, the flow field in the channel is not strictly parallel to the channel wall. There are velocity components v and w in the cross sectional plane, though they are small in comparison with u, due to the entrance effect, the secondary flow generated in the bend [27], the flow generated by Joule heating [19–20], and the local unsteady flow associated with rupture of cells. These transverse flows, especially the last two, are helpful for effective cell lysis in the present continuous flow-through device as discussed in Section 4.2 of the paper. However, Equations (S3a), (S3b) and (S5) provide us adequate estimations, at least of the correct order of magnitudes, for the mechanical shear stresses contributed by the fluid flow in the device.

(II) Electric Field and Maxwell Stress Tensor

The electric field, and thus the Maxwell stress tensor, is periodic along the axis of the channel for most of the region in the device except near the inlet and the bend in Figure 1b of the paper. The electric potential in the fluid medium (with electrical conductivity ≈ 1 S/m here) is governed by the Laplace Equation [28], and was solved in the region $0 \le x \le \lambda$, $0 \le y \le a$, and $0 \le z \le b$ in Figure 1b, subject to specified ac potentials on the electrodes, insulated boundary conditions at the glass (electrical conductivity ≈ 10^{-15}–10^{-11} S/m) and PDMS (electrical conductivity ≈ 10^{-15}–10^{-11} S/m) walls, and periodic condition along the x-direction. Such a geometric configuration and boundary conditions suggest that the electric potential, Ψ, to be two-dimensional in the xz-plane and varying with time, t, and can be written as

$$\Psi(x,z,t) = \Phi(x,z)\cos(\omega t), \quad \text{(S6)}$$

with $\Phi(x,z)$ obtained by solving

$$\nabla^2\Phi(x,z) = 0, \quad \text{(S7)}$$

subject to

$$\Phi(x,0) = -V_0, \quad \text{for} \quad 0 \le x \le d/2, \quad \text{and} \quad 3d/2 + 2s \le x \le 2d + 2s, \quad \text{(S7a)}$$

$$\Phi(x,0) = V_0, \quad \text{for} \quad d/2 + s \le x \le 3d/2 + s, \quad \text{(S7b)}$$

$$\frac{\partial \Phi}{\partial z}(x,0) = 0 \quad \text{for} \quad d/2 \le x \le d/2 + s \quad \text{and} \quad 3d/2 + s \le x \le 3d/2 + 2s, \quad \text{(S7c)}$$

$$\frac{\partial \Phi}{\partial z}(x,b) = 0 \quad \text{for} \quad 0 \le x \le 2d + 2s, \quad \text{(S7d)}$$

and

$$\Phi(0,z) = \Phi(2d + 2s, z) \quad \text{for} \quad 0 \le z \le b. \quad \text{(S7e)}$$

Equation (S7) with boundary conditions (S7a–e) were solved numerically using finite difference approximation and the Gauss-Seidel iterative method. With electric potential known, the electric field, $\mathbf{E}(x,z,t)$, can be calculated through

$$\mathbf{E}(x,z,t) = E_x\mathbf{e}_x + E_z\mathbf{e}_z = -\nabla\Psi = -\nabla\Phi\cos(\omega t) = -\frac{\partial\Phi}{\partial x}\cos(\omega t)\mathbf{e}_x - \frac{\partial\Phi}{\partial z}\cos(\omega t)\mathbf{e}_z$$
$$= \mathbf{E}_\mathrm{p}\cos(\omega t) = E_\mathrm{px}\cos(\omega t)\mathbf{e}_x + E_\mathrm{pz}\cos(\omega t)\mathbf{e}_z, \quad \text{(S8)}$$

with $\mathbf{e}_x$ and $\mathbf{e}_z$ the unit vectors along the x and z directions, respectively. The corresponding electric field components E_x and E_z are time varying functions in the ac field, and their time averages are zero. We will examine the electric field phasor components, E_px and E_pz, and the root mean square of the electric field,

$$E_\mathrm{rms} = \sqrt{E_x^2 + E_z^2} = \sqrt{\frac{\omega}{2\pi}\int_0^{2\pi/\omega}(E_x^2 + E_z^2)dt} = \frac{1}{\sqrt{2}}\sqrt{\left(-\frac{\partial\Phi}{\partial x}\right)^2 + \left(-\frac{\partial\Phi}{\partial z}\right)^2} = \frac{1}{\sqrt{2}}\sqrt{E_\mathrm{px}^2 + E_\mathrm{pz}^2}, \quad \text{(S9)}$$

where the "mean" refers to the average over time for one period ($2\pi/\omega$) of electric field variation.

The Maxwell stress, expressed in index form [24], is

$$T_{ij} = \varepsilon_0\varepsilon_r E_i E_j - \frac{1}{2}\varepsilon_0\varepsilon_r\delta_{ij}E_k E_k, \quad \text{(S10)}$$

where $\varepsilon_0 = 8.854\times10^{-12}\mathrm{m^{-3}kg^{-1}s^4A^2}$ is the permittivity in vacuum, ε_r is the relative permittivity of the medium, δ_{ij} is the Kronecker delta, indices i or j stand for x, y or z, and $E_k E_k = E_x^2 + E_y^2 + E_z^2$. The time mean Maxwell stress tensor over a period of electric excitation of the present electric field, expressed in matrix form, is

$$\mathbf{T} = \begin{bmatrix} T_{xx} & T_{xy} & T_{xz} \\ T_{yx} & T_{yy} & T_{yz} \\ T_{zx} & T_{zy} & T_{zz} \end{bmatrix} = \varepsilon_0\varepsilon_r \begin{bmatrix} 0.25\left(E_\mathrm{px}^2 - E_\mathrm{pz}^2\right) & 0 & 0.5E_\mathrm{px}E_\mathrm{pz} \\ 0 & -0.25\left(E_\mathrm{px}^2 + E_\mathrm{pz}^2\right) & 0 \\ 0.5E_\mathrm{pz}E_\mathrm{px} & 0 & 0.25\left(E_\mathrm{pz}^2 - E_\mathrm{px}^2\right) \end{bmatrix}. \quad \text{(S11)}$$

We have stress components T_{xx}, T_{zz} and T_{xz} on the xz-plane, as well as a compressive component T_{yy}, for the present two-dimensional electric field in the xz-plane.

The stress components change with the orientation of the surface they applied. The principal stresses (extreme stresses) at a point among different orientations of the surface they exerted are obtained by solving [29]

$$T_\mathrm{p}^3 - I_1T_\mathrm{p}^2 + I_2T_\mathrm{p} - I_3 = 0, \quad \text{(S12)}$$

where

$$I_1 = T_{xx} + T_{yy} + T_{zz}, \quad \text{(S12a)}$$

$$I_2 = T_{xx}T_{yy} + T_{yy}T_{zz} + T_{zz}T_{xx} - T_{yz}T_{zy} - T_{zx}T_{xz} - T_{xy}T_{yx}, \quad \text{(S12b)}$$

and

$$I_3 = T_{xx}T_{yy}T_{zz} + T_{xy}T_{yz}T_{zx} + T_{xz}T_{yx}T_{zy} - T_{xz}T_{yy}T_{zx} - T_{xx}T_{yz}T_{zy} - T_{xy}T_{yx}T_{zz} \quad \text{(S12c)}$$

are three invariants of the tensor. The three solutions are

$$T_\mathrm{p1} = \frac{I_1}{3} + \frac{2}{3}\sqrt{I_1^2 - 3I_2}\cos\phi, \quad \text{(S13a)}$$

$$T_{p2} = \frac{I_1}{3} + \frac{2}{3}\sqrt{I_1^2 - 3I_2}\cos\left(\phi - \frac{2\pi}{3}\right), \tag{S13b}$$

and

$$T_{p3} = \frac{I_1}{3} + \frac{2}{3}\sqrt{I_1^2 - 3I_2}\cos\left(\phi - \frac{4\pi}{3}\right), \tag{S13c}$$

where

$$\cos 3\phi = \frac{2I_1^3 - 9I_1I_2 + 27I_3}{2\left(I_1^2 - 3I_2\right)^{3/2}}. \tag{S14}$$

For the time mean Maxwell stress components shown in Equation (S11),

$$I_1 = -\frac{\varepsilon_0\varepsilon_r}{4}\left(E_{px}^2 + E_{pz}^2\right) = -\frac{\varepsilon_0\varepsilon_r}{2}E_{rms}^2, \quad I_2 = -I_1^2, \quad I_3 = -I_1^3, \tag{S15a}$$

$$\cos 3\phi = \mp 1 \text{ or } \phi = \frac{\pi}{3} \text{ or } 0, \quad \text{and} \quad \Delta \equiv \sqrt{I_1^2 - 3I_2} = \pm 2\left|I_1\right|. \tag{S15b}$$

It follows that

$$T_{p1} = \frac{|I_1|}{3},\ T_{p2} = \frac{|I_1|}{3},\ T_{p3} = -\frac{5|I_1|}{3},\ T_{1\text{-}2} = 0,\ T_{1\text{-}3} = |I_1|,\ T_{2\text{-}3} = |I_1| \text{ if } \phi = \frac{\pi}{3} \text{ and } \Delta = 2|I_1|. \tag{S16a}$$

$$T_{p1} = \frac{-4|I_1|}{3},\ T_{p2} = \frac{-4|I_1|}{3},\ T_{p3} = |I_1|,\ T_{1\text{-}2} = 0,\ T_{1\text{-}3} = \frac{7|I_1|}{6},\ T_{2\text{-}3} = \frac{7|I_1|}{6} \text{ if } \phi = \frac{\pi}{3} \text{ and } \Delta = -2|I_1|. \tag{S16b}$$

$$T_{p1} = |I_1|,\ T_{p2} = \frac{-4|I_1|}{3},\ T_{p3} = \frac{-4|I_1|}{3},\ T_{1\text{-}2} = \frac{7|I_1|}{6},\ T_{1\text{-}3} = \frac{7|I_1|}{6},\ T_{2\text{-}3} = 0 \text{ if } \phi = 0 \text{ and } \Delta = 2|I_1|. \tag{S16c}$$

$$T_{p1} = \frac{-5|I_1|}{3},\ T_{p2} = \frac{|I_1|}{3},\ T_{p3} = \frac{|I_1|}{3},\ T_{1\text{-}2} = |I_1|,\ T_{1\text{-}3} = |I_1|,\ T_{2\text{-}3} = 0 \text{ if } \phi = 0 \text{ and } \Delta = -2|I_1|. \tag{S16d}$$

Here the magnitudes of the maximum shear stresses $T_{1-2} = \left|T_{p1} - T_{p2}\right|$, $T_{1-3} = \left|T_{p1} - T_{p3}\right|$ and $T_{2-3} = \left|T_{p2} - T_{p3}\right|$. Among the four solutions in Equations (S16a–d), we pick the case (i.e., Equation (S16b) or (S16c)) with the maximum value of the shear stress, and denote it as T_s, with the corresponding principal stresses denoted by T_{tensile} (for tensile stress) and $T_{\text{compressive}}$ (for compressive stress). The final results are

$$T_{\text{tensile}} = \frac{1}{2}\varepsilon_0\varepsilon_r E_{rms}^2, \quad T_{\text{compressive}} = -\frac{2}{3}\varepsilon_0\varepsilon_r E_{rms}^2 \quad \text{and} \quad T_{\text{shear}} = \frac{7}{12}\varepsilon_0\varepsilon_r E_{rms}^2. \tag{S17}$$
